# Exploring Reasons for Delayed Start-of-Care Nursing Visits in Home Health Care: Algorithm Development and Data Science Study

**DOI:** 10.2196/31038

**Published:** 2021-12-30

**Authors:** Maryam Zolnoori, Jiyoun Song, Margaret V McDonald, Yolanda Barrón, Kenrick Cato, Paulina Sockolow, Sridevi Sridharan, Nicole Onorato, Kathryn H Bowles, Maxim Topaz

**Affiliations:** 1 School of Nursing Columbia University New York, NY United States; 2 Center for Home Care Policy & Research Visiting Nurse Service of New York New York, NY United States; 3 College of Nursing and Health Professions Drexel University Philadelphia, PA United States; 4 Center for Transitions and Health School of Nursing University of Pennsylvania Philadelphia, PA United States

**Keywords:** delayed start-of-care nursing visit, home healthcare services, natural language processing, nursing note, NLP, nursing, eHealth, home care, clinical notes, classification, clinical informatics

## Abstract

**Background:**

Delayed start-of-care nursing visits in home health care (HHC) can result in negative outcomes, such as hospitalization. No previous studies have investigated why start-of-care HHC nursing visits are delayed, in part because most reasons for delayed visits are documented in free-text HHC nursing notes.

**Objective:**

The aims of this study were to (1) develop and test a natural language processing (NLP) algorithm that automatically identifies reasons for delayed visits in HHC free-text clinical notes and (2) describe reasons for delayed visits in a large patient sample.

**Methods:**

This study was conducted at the Visiting Nurse Service of New York (VNSNY). We examined data available at the VNSNY on all new episodes of care started in 2019 (N=48,497). An NLP algorithm was developed and tested to automatically identify and classify reasons for delayed visits.

**Results:**

The performance of the NLP algorithm was 0.8, 0.75, and 0.77 for precision, recall, and F-score, respectively. A total of one-third of HHC episodes (n=16,244) had delayed start-of-care HHC nursing visits. The most prevalent identified category of reasons for delayed start-of-care nursing visits was no answer at the door or phone (3728/8051, 46.3%), followed by patient/family request to postpone or refuse some HHC services (n=2858, 35.5%), and administrative or scheduling issues (n=1465, 18.2%). In 40% (n=16,244) of HHC episodes, 2 or more reasons were documented.

**Conclusions:**

To avoid critical delays in start-of-care nursing visits, HHC organizations might examine and improve ways to effectively address the reasons for delayed visits, using effective interventions, such as educating patients or caregivers on the importance of a timely nursing visit and improving patients’ intake procedures.

## Introduction

Over the last several decades in the United States, recognition has been growing of the importance of post–acute care settings, including long-term care hospitals, inpatient rehabilitation facilities, and skilled nursing facilities [1,2]. One specific type of post–acute care setting, home health care (HHC), has been growing rapidly; according to the Centers for Medicare & Medicaid Services (CMS), the expenditure on HHC was over US $102 billion in 2018, which indicated a more than 30% increase compared to 5 years ago.[3] Currently, more than 12,000 HHC agencies in the United States serve more than 6 million patients annually [4]. In addition, the United States and global populations are becoming older and suffering from more complex health conditions that require care in the community [5,6]; hence, the demand for HHC services is projected to increase incrementally in the following decades.

HHC consists of intermittent home visits conducted by skilled health care providers (eg, registered nurses, physical therapists, social workers). Currently, over 6 million patients are admitted to more than 12,000 HHC agencies across the United States annually [7-9]. Despite national and local efforts for quality improvement, approximately 1 in 5 HHC patients are hospitalized or visit the emergency department during their HHC episode [7,10]. Up to two-thirds of these hospitalizations occur within the first 2 weeks of HHC services [11-15]. A significant portion of hospitalizations and emergency department visits from HHC may be prevented by timely and appropriately targeted home care services [13,15-17].

The start-of-care nursing visit is one of the most critical steps of the HHC episode [18,19]. This visit includes medication reconciliation, patient self-care capability assessment, home environment examination, and discussion regarding caregiver availability and ability to help. Based on this evaluation, a unique care plan is created [20]. Hence, appropriate timing of the first visit is crucial.

The CMS requires agencies to conduct a start-of-care visit within 48 hours of HHC referral or within 48 hours of the patient’s return home [21]. However, we found about one-third of patients admitted to a large HHC agency were seen later than 48 hours post–hospital discharge date in our ongoing study focused on identifying patients at risk for poor outcomes during the hospital to HHC transition (R01-NR018831). Delays in the initiation of HHC services may have a profound negative impact on patients’ outcomes. The majority of patients who are admitted to HHC are vulnerable older adults who are discharged from hospitals with chronic and acute medical conditions such as advanced heart failure, acute coronary syndrome, and diabetes [4]. Many of these patients need immediate access to care to avoid negative outcomes; studies have shown that avoiding delays in the start of care among HHC patients can improve functional and physiological outcomes [22] and reduce the risk of hospitalization and emergency department visits by up to 50% [23].

No previous study has investigated why start-of-care HHC nursing visits are delayed. One explanation may be that most delayed visit reasons are documented in free-text HHC nursing notes. Given a large volume of clinical notes (~100,000), manual review is not feasible, calling for the use of data science methods.

Fortunately, recent data science analytical techniques can extract insights from free-text documentation. Specifically, natural language processing (NLP) algorithms can be applied to large collections of clinical documents to identify a diverse range of concepts and extract meaning from documents [24]. Often, NLP algorithms rely on manually curated, rule-based vocabularies generated by subject matter experts [24].

An example of NLP algorithm use is the identification of social risk factors among patients discharged from hospitals using discharge summaries [24]. The researchers (including physicians, nurses, and pharmacologists) first developed comprehensive vocabularies of words and expressions that describe several key social risk factor categories including tobacco use, drug abuse, depression, and housing instability. Words and expressions representing the concept of drug abuse include amphetamine abuse, cocaine abuse, and intravenous drug use. NLP was used to search discharge summaries (n=~100,000) to find the manually curated, rule-based terms related to 1 or more social risk factor categories.

In HHC settings, previous applications of NLP focused on identifying patients who experienced falls [25], dementia symptoms [26], and hospitalization risk factors [27]. This study demonstrates the application of NLP to analyze reasons for delayed start-of-care HHC nursing visits. Specifically, we aimed to (1) develop and test an NLP algorithm that automatically (without human involvement) identifies reasons for potential delay in timing of the start-of-care HHC nursing visit in free-text clinical notes and (2) use descriptive statistics to describe the identified reasons for delayed start-of-care HHC nursing visits in a large patient sample.

## Methods

### Study Setting

This study was conducted at Visiting Nurse Service of New York (VNSNY), the largest not-for-profit urban home care agency in the United States. The study research protocol was approved by the institutional review board of the VNSNY in October 2019.

### Study Sample

The study sample comprises all HHC patients admitted during the calendar year of 2019 following a hospitalization. The patients’ status of hospitalization prior to HHC was extracted from the M1000_3 variable on the Outcome and Assessment Information Set (OASIS), a patient-specific, standardized assessment required under Medicare HHC rules [28]. The study sample comprises 45,390 admitted patients, some of whom were admitted more than once, resulting in 48,497 HHC episodes. CMS reimbursement for an HHC episode in 2019 was for 60 days of care. As we were interested in understanding all reasons for delayed start-of-care nursing visits, we conducted this analysis at the HHC episode level.

Dates of hospital discharge and start-of-care HHC nursing visit were extracted from the OASIS. We calculated the difference in days between the following OASIS items: M1005: Inpatient Discharge Date and M0090: Date Assessment Completed. This study defined delayed start-of-care HHC nursing visits as visits conducted more than 2 days after hospital discharge. Approximately one-third (16,244/48,497, 33.5%) of episodes met this criterion.

### Documentation Categories for Documenting Delayed Start-of-Care

The electronic health record (EHR) had a standardized option that allowed HHC staff to document reasons for delayed visits. The standardized categories included no answer at the door or phone, administrative or scheduling issues, and patient/family request to postpone or refuse some HHC services.

### Structure of Clinical Notes With Delayed Start-of-Care

We extracted 118,767 clinical notes documented for HHC episodes with delayed visits. More than 1 note could be documented for an episode. There were 22 note types with the most common being narrative notes that included nursing and other HHC admission staff comments (23,753/118,767, 20%), initial welcome call notes that described the initial welcome telephone call to introduce HHC services to the patient (n=20,190, 17%), intake clinical comment notes that provided clinical details of the admitted patients (n=14,252, 12%), insurance and other additional information notes with information on patient insurance and additional clinical factors (eg, list of medications; n=9501, 8%), telephone communication notes describing any phone contact between HHC staff and patients or family (n=4750, 4%), and 17 other less frequent note categories (n<3563, <3% each).

### Natural Language Processing for Analysis of Clinical Notes with Delayed Start-of-Care

We developed an NLP algorithm using a regular expression technique [24] where algorithmic rules were crafted to match certain language patterns describing reasons for delayed visits. Regular expression is a powerful text search technique that uses alphabetic characters, numeric expressions, and nonalphanumeric expressions. The regular expression approach can help find certain predefined lists of keywords in the texts [29]. The goal is to capture as much lexical variation as possible using keywords and language patterns.

As the first step of developing the NLP algorithm, the data preprocessing steps involved lowercasing, stripping (removing extra spaces), and removing special characters (eg, [, \, ^, $, |, ?, *, +, (, ), ]). We did not remove stop words because they were important for identifying the reasons for delayed start-of-care. For example, negation indicators (such as no and not) or auxiliary words (such as do, have, and has) were important for identifying if a patient requested to postpone the HHC services. We also did not perform lemmatization or stemming because keeping the original form of words, particularly the verbs, was important for identifying a pattern of late visit.

As the second step of developing the NLP algorithm, we created regular expression rules to capture language describing patient or family requests to postpone HHC or refuse some HHC services. This included the following phrases: “pt declined VNS visit today,” “patient refused visit and asks for visit tomorrow,” “daughter asks to reschedule SOC for Friday.” These phrases have some word patterns, such as “patient/pt/family refused,” “asks for visit tomorrow,” and “reschedule SOC for Friday,” that were used to develop regular expression rules. Table 1 provides more examples of regular expressions applied to identify reasons for delayed start-of-care HHC nursing visits in clinical notes. Multimedia Appendix 1 provides examples of regular expression syntaxes used for identifying patterns of delayed start-of-care for the specified categories.

**Table 1:** Examples of regular expressions applied to identify reasons for delayed start-of-care HHC^a^ nursing visits in clinical notes.

Category and examples of regular expressions			Example from a clinical note^b^
**No answer at the door or phone**			
	Unable to leave VM^c^; unable to leave MSG^d^	“SOC attempted. *Unable to leave VM* to confirm visit, mailbox full, *unable to leave MSG*”	
	Unable to reach; contact numbers are incorrect	“*Unable to reach patient, contact numbers listed are incorrect.*”	
	No response; patient not found	“Multiple phone calls and walk by attempts and *no response, patient not found*”	
**Patient/family request to postpone or refuse some HHC services**			
	Patient declined	“*Patient declined* SOC today”	
	Request to schedule SOC^e^ for tomorrow	“Caregiver and daughter informed VN that pt will not be available today and made the *request to reschedule SOC for tomorrow*”	
**Administrative or scheduling issues**			
	Case is pending	“Request for VN visit for wound care clinical triage needed, *case is pending admin approval*.”	
	Missing information	“Intake referral inquiry, subject: FW: *missing information,* patient is missing attachment or referral source.”	
	Visiting nurse not available	“SOC visit rescheduled for tomorrow. *VN unable to make scheduled appt. time*. Rescheduled”	

^a^HHC: home health care.

^b^Italicized text denotes the identified language in the clinical notes that indicates reasons for delayed start-of-care HHC nursing visits.

^c^VM: voice message.

^d^MSG: message.

^e^SOC: start-of-care.

### Evaluation of the NLP algorithm

To evaluate the accuracy of our NLP algorithm, we extracted 100 clinical notes from each of the 22 note types, resulting in 2200 clinical notes. Each note was reviewed independently by 3 HHC experts, who labeled the note with 1 or more of the 3 delayed start-of-care HHC nursing visit categories, where applicable. The experts included a PhD-prepared registered nurse, a social worker, and an experienced research analyst. There was a high level of initial interrater agreement between the experts (κ>0.8) [30]. Disagreements were resolved via expert team discussion.

We applied our NLP algorithm on the testing set and calculated the accuracy of NLP in identifying reasons for delayed visits. For each category of delayed visit reasons, we calculated the precision (defined as the number of true positives out of the total number of predicted positives returned by the NLP algorithm), recall (the number of true positives out of the actual number of positives), and F-score (the weighted harmonic mean of the precision and recall). These metrics vary between 0 and 100%, with higher values indicating better NLP algorithm concept identification performance; values above 75% indicate high performing NLP algorithms.

## Results

### Cohort Demographic

Table 2 provides a summary of the demographics of patients admitted to VNSNY.

The majority of patients admitted to VNSNY were aged over 65 years (29,985/48,497, 61.8%). Females comprised a larger fraction of the patient population compared to males. The proportion of non-Hispanic White patients was significantly higher than that of other races. Hypertension (28,804/48,497, 59.4%) was the most common disease among patients, followed by diabetes (n=15,204, 31.4%) and cancer (n=7591, 15.7%). The majority of the patients (36,106/48,497, 74.5%) could receive support from their family members or their caregivers.

**Table 2:** Demographics of patients admitted to the Visiting Nurse Service of New York (VNSNY).

Characteristic			Patients (N=48,497), n (%)
**Demographics**			
	**Age (years)**		
		<65	18,512 (38.2)
		≥65	29,985 (61.8)
	**Sex**		
		Female	28,094 (57.9)
		Male	20,403 (42.1)
	**Race**		
		Non-Hispanic White	21,070 (43.5)
		Non-Hispanic Black	11,574 (23.9)
		Hispanic	11,416 (23.5)
		Other	4437 (9.2)
**Type of insurance**			
	Dual eligibility		5777 (11.9)
	Medicare FFS^a^ only		13,147 (27.1)
	Medicaid FFS only		420 (0.9)
	Any HMO^b^		15,954 (32.9)
	Other (eg, private)		13,199 (27.2)
**Living situation**			
	Living with others (eg, congregate living)		36,106 (74.5)
	Living alone		12,391 (25.6)
**Current condition**			
	Congestive heart failure		6682 (13.8)
	Cardiac arrhythmias		4882 (10.1)
	Hypertension		28,804 (59.4)
	Chronic pulmonary disease		6678 (13.8)
	Diabetes		15,204 (31.4)
	Renal failure		5244 (10.8)
	Cancer		7591 (15.7)

^a^FFS: fee-for-service.

^b^HMO: health maintenance organization.

### Measuring Performance of the NLP algorithm

The overall accuracy of NLP in detecting language related to reasons of delayed visits was high, as reflected by the total F-score of 0.77 (Table 3). When applied to all clinical notes in the study (n=118,767), the NLP algorithm identified 21,433 reasons for delayed visits documented in 20,536 clinical notes for 10,644 unique HHC episodes.

Although the total F-score of the NLP algorithm for identifying the reason of delayed start-of-care is promising, it also implies that the algorithm could not capture all variations for delayed start-of-care in clinical notes. For example, it did not identify the following notes indicating that the patient postponed the start-of-care: (1) “Pt will return call from [nurse], he prefers the soc after 3 (PM),” (2) “Spoke with patient’s caregivers, stated that they are moving to a new apartment and the patient would like to have soc in another time.”

**Table 3:** Precision, recall, and F-score of the natural language processing algorithm to identify categories of delayed visit reasons. Scores above 0.75 indicate good performance.

Delayed visit reason category	Precision	Recall	F-score
No answer at the door or phone	0.73	0.81	0.76
Administrative or scheduling issues	﻿﻿0.85	0.71	0.77
Patient or family request to postpone or refuse some home health care services	﻿0.81	0.73	0.77
Mean	0.8	0.75	0.77

### Prevalence of Delayed Start-of-Care HHC Visits in Standardized Documentation

In total, 16,244 HHC episodes had a delayed start-of-care HHC visit. Among all 16,244 episodes with a delayed start-of-care nursing visit, 3097 (19.1%) had reasons for delayed start-of-care documented using standardized documentation. Of the 3097 cases with documentation, 5484 unique reasons for delayed start-of-care HHC nursing visits were found (a single HHC episode could have more than 1 reason documented).

### Reasons for Delayed Start-of-Care Within Clinical Notes

**Figure 1:**
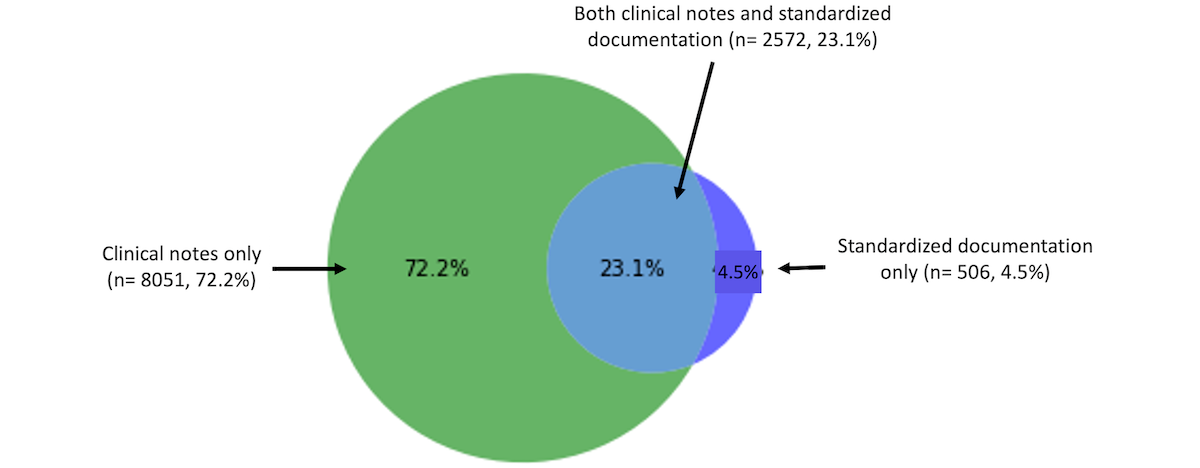
Sources of documented reasons for delayed start-of-care HHC nursing visits.

Overall, 72.2% of the delayed visit reasons (8051/11,129, Figure 1) were documented in clinical notes. When the NLP algorithm results and the standardized documentation categories were combined, reasons for delayed visits were identified for 11,148 of the 16,244 HHC episodes (68.6%). We found that 23.1% (2572/11,129) of delayed visit reasons were documented in both documentation sources (Figure 1), while only 4.5% (n=506) of the reasons were documented in standardized documentation only. For the remaining 31.4% HHC episodes (5096/16,244), no delayed visit reasons were identified.

The most prevalent categories of reasons for delayed start-of-care HHC nursing visits identified by the NLP algorithm were “no answer at the door or phone” (3728/8051, 46.3%), followed by “patient/family request to postpone or refuse some HHC services” (n=2858, 35.5%) and “administrative or scheduling issues” (n=1465, 18.2%).

**Figure 2:**
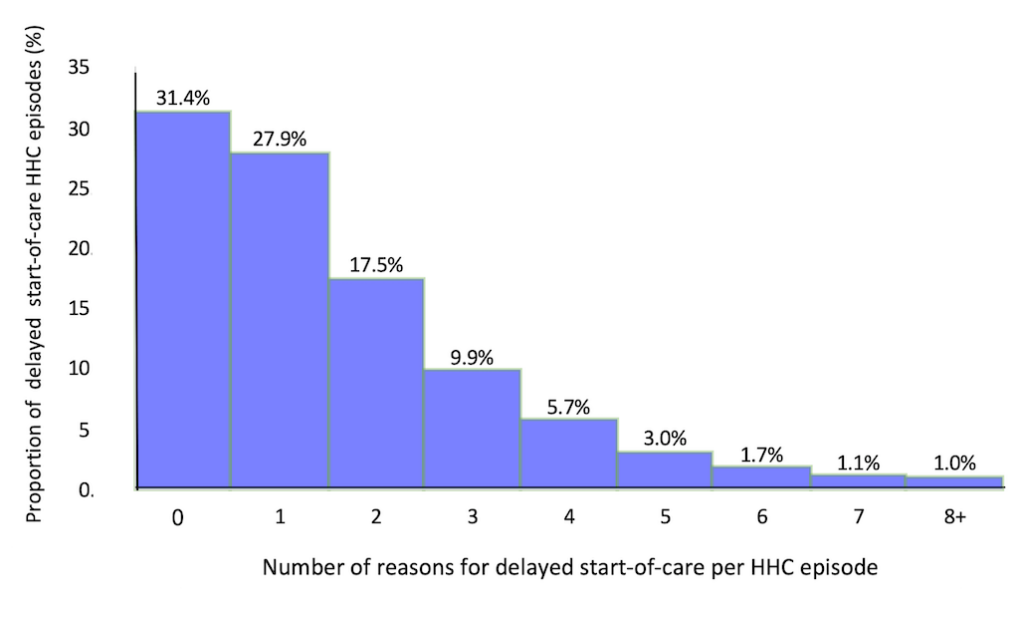
Number of reasons for delayed start-of-care home health care (HHC) nursing visits per home health care episode.

Further analysis showed that in more than 40% of episodes with delayed start-of-care HHC nursing visits, 2 or more reasons were documented. Figure 2 shows that 17.5% (1409/8051) of the HHC episodes had 2 reasons for delayed start-of-care, 9.9% (794/8051) of HHC episodes had 3 reasons, and 5.7% (459/8051) of the HHC episodes had 4 reasons.

**Figure 3:**
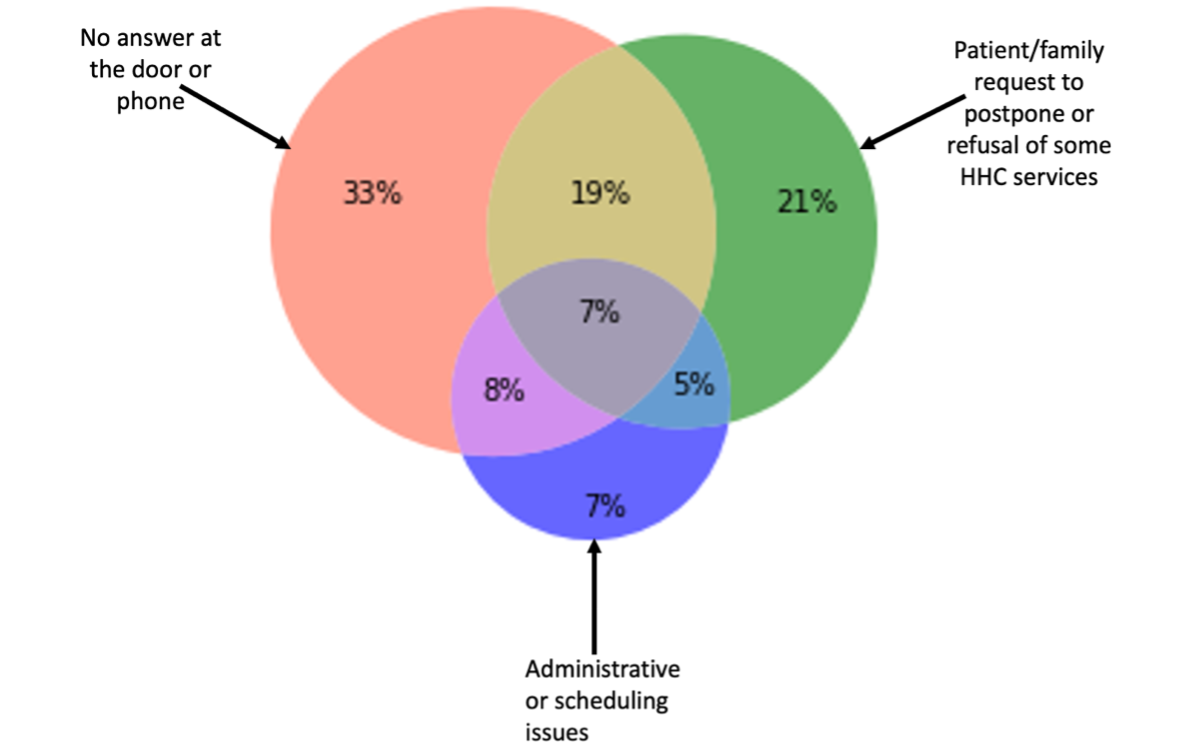
Among cooccurring reasons for delayed start-of-care, overlap of reasons for delayed start-of-care home health care (HHC) nursing visits.

The analysis of cooccurrence of reasons for delayed start-of-care HHC nursing visits (Figure 3) indicated that 19% (1529/8051) of HHC episodes with documented delayed visit reasons had both “no answer at the door or phone” and “patient/family request to postpone or refuse some HHC services”; 8% (644/8051) of HHC episodes had both “no answer at the door or phone” and “administrative or scheduling issues.” All 3 reasons were documented for 7% (563/8051) of HHC episodes. 

## Discussion

### Principal Findings

This is the first study we are aware of that examines reasons for delayed start-of-care HHC nursing visits in a large population of HHC patients. Identifying reasons for delayed start-of-care is critical due to the association between delayed care and negative outcomes. The majority of patients requiring HHC are clinically complex and vulnerable older adults who were discharged from hospitals with a need for immediate post–acute care [31]. Delayed start-of-care may increase the risk of complications, emergency department visits, hospitalizations, and even death [32].

The findings of this study indicate that when a reason is provided, most reasons for delayed start-of-care HHC nursing visits (8051/11,129, 72.2%) are documented exclusively in narrative clinical notes. Large-scale analysis of thousands of clinical notes can now be accomplished via innovative data science methods, including NLP.

We developed and tested an NLP algorithm to identify patterns of documented delayed start-of-care HHC nursing visit reasons in clinical notes. The NLP algorithm achieved high accuracy in identifying documented reasons when tested on an expert-reviewed sample of clinical notes. NLP algorithms have a wide range of potential applications in extracting meaningful patterns from EHR clinical notes [33]. We believe that NLP algorithms will be integrated into HHC EHRs in the future to process ongoing clinical documentation. Such integration can help HHC managers and practicing nurses to identify and address reasons for delayed start-of-care HHC nursing visits, potentially reducing delays in HHC services and improving patient outcomes.

Our findings indicate that the most prevalent category of delayed start-of-care HHC nursing visits was “no answer at the door or phone.” Potential explanations include having incorrect phone or address information transmitted to the HHC organization, last minute decisions by patients to recover in a location other than their own home, and a lack of communication between the HHC organization and the patient about the patient’s availability. To address this issue, referring hospital clinicians may need to discuss the timing and importance of the first visit with the patient and family and obtain accurate contact information. In addition, it may be necessary to develop a clear communication plan with the HHC agency in the process of transferring the patient from the hospital to the HHC setting.

Previous studies [34-42] and our anecdotal experience show that in reality, very little information is available to HHC nurses about newly admitted patients. Usually, HHC referrals from hospitals or primary care providers include administrative information, such as patients’ insurance status and billing address. It may be that patient contact information indicated in referrals is not always accurate, hence the delays in care due to phone calls placed to incorrect or outdated phone numbers or visits to outdated addresses. A recent report explored the availability of address information in a large hospital system and found that for 99% of patients (~2.1 million patients), a billing address was documented [43]. However, if patients were located at another address, the documentation of an alternative address was missing for 99.7% of these patients. Further investigation of the accuracy of the referral address and phone information might shed light on some of the interventions that can help reduce delayed care.

The second most prevalent reason for delayed start-of-care HHC nursing visits was “patient/family request to postpone or refuse some HHC services.” Although some related care delays are probably unavoidable, referring physicians and HHC clinicians may need to spend more time articulating the importance of start-of-care HHC nursing visits. Several recent studies indicate many patients may not understand the value of HHC and refuse care. A recent national study found that more than half of the patients referred to HHC from hospitals never receive HHC services [44]. Another study found that about 1 in 3 patients referred to post–acute health services, including HHC, refuse care [18]. The patients who refused post–acute care had twice higher odds of hospitalization or emergency department visits compared to patients with timely visits. Increased patient understanding of the importance of HHC services might help to resolve some of the issues related to this prevalent reason for delayed start-of-care HHC nursing visits.

In about 20% of HHC episodes, delayed start-of-care was due to “administrative or scheduling issues.” Some of the specific issues were inadequate or incomplete insurance coverage, lack of forms and documents, and a lack of available HHC nurses to complete a start-of-care visit on time. To resolve these issues, HHC organizations may need to develop better and more streamlined patient intake procedures and identify several ways to adjust schedules when a nurse calls in sick or is otherwise unavailable to take on new cases.

Our results indicate that in more than 40% of HHC episodes, there were 2 or more reasons for delayed start-of-care HHC nursing visits. The most common cooccurring reasons indicated in about 20% of HHC episodes were “no answer at the door or phone” and “patient/family request to postpone or refuse some HHC services.” In a relatively small number of cases (7%), patients had a combination of all 3 reasons for delayed start-of-care HHC nursing visits. We hypothesize that patients with cooccurring reasons might be more likely to have a delayed start-of-care HHC nursing visit due to the challenges of addressing all reasons of delays in this group of patients. Clinicians may decide to prioritize patients with cooccurring reasons for appropriate interventions to reduce the likelihood of delayed start-of-care. The patient’s demographic and clinical factors might affect their risk for a delayed start-of-care HHC nursing visit. Identifying the relationship between these factors and reasons for delayed visits may help clinicians target patients at high risk for delayed visits with specific interventions. This is an area that requires further investigation.

### Implications for Health Care Administrators

The findings of this study provide insight into the application of NLP techniques to identify reasons for delayed start-of-care in HHC agencies and potential interventions to address the delays and avoid subsequent negative outcomes. HHC administrators may begin considering the use of NLP techniques to analyze the HHC nursing visits for patients that were seen later than 48 hours post–hospital discharge date to identify the reasons for delays and potential interventions. Obtaining and maintaining accurate patient contact information, educating patients about the importance of timely HHC services, and improving the procedures of patient intake and the scheduling system are among the interventions we suggested to reduce the risk of delayed start-of-care due to the following reasons, respectively: “no answer at the door or phone,” “patient/family request to postpone or refuse some home health care services,” and “administrative or scheduling issues.”

### Limitations

The findings of this study should be considered in light of several limitations. First, the data set was from a single HHC agency located in the northeast United States. Although the HHC agency is large, its services are limited to individuals residing in a particular catchment area. Therefore, the reasons for delayed visits may not be generalizable to HHC agencies in other locations. Second, the available data did not have information on the hour of discharge from the hospital, so the analysis was based on days, not hours as in the CMS regulations. Third, the NLP algorithms developed in this study had a total precision of 0.8 and recall of 0.75. These findings indicate that some reasons for delayed start-of-care HHC nursing visits identified by NLP were incorrect due to algorithm errors. It is unclear if additional reasons would have been identified if more information on these episodes was provided.

### Conclusions

To avoid delays in critical start-of-care nursing visits, HHC agencies and hospitals should consider examining and improving accurate patient or family contact information collection. In addition, HHC agencies should consider providing targeted education about the importance of the early initiation of HHC services to reduce patient and family refusals and/or requests to postpone care. Finally, HHC agencies should make efforts to reduce administrative and scheduling issues to enable timely and effective care.

## Appendix

Multimedia Appendix 1 Examples of regular expression rules developed to identify reasons for delayed start-of-care home health care nursing visits in clinical notes.

## Abbreviations

CMS: Centers for Medicare & Medicaid Services
EHR: electronic health record
HHC: home health care
NINR: National Institute of Nursing Research
NLP: natural language processing
OASIS: Outcome and Assessment Information Set
VNSNY: Visiting Nurse Service of New York

## References

[1] Jarvis WR (2001). Infection control and changing health-care delivery systems. Emerg Infect Dis.

[2] Hardin L, Mason DJ (2019). Bringing it home: the shift in where health care is delivered. JAMA.

[3] Hartman M, Martin AB, Benson J, Catlin A (2020). National health care spending in 2018: growth driven by accelerations in Medicare and private insurance spending. Health Aff (Millwood).

[4] United States Centers for Disease Control and Prevention (2016). Home health care. Centers for Disease Control and Prevention.

[5] Mitzner TL, Beer JM, McBride SE, Rogers WA, Fisk AD (2009). Older adults' needs for home health care and the potential for human factors interventions. Proc Hum Factors Ergon Soc Annu Meet.

[6] Landers S, Madigan E, Leff B, Rosati R, McCann B, Hornbake R, MacMillan R, Jones K, Bowles K, Dowding D, Lee T, Moorhead T, Rodriguez S, Breese E (2016). The future of home health care: a strategic framework for optimizing value. Home Health Care Manag Pract.

[7] The Medicare Payment Advisory Commission (2018). March 2018 report to the congress: medicare payment policy. MedPAC.

[8] (2010). Basic statistics about home care: updated 2010. National Association for Home Care & Hospice.

[9] The Medicare Payment Advisory Commission (2018). Home health care services. MedPAC.

[10] U.S. Centers for Medicare and Medicaid Services Medicare.gov.

[11] Zolnoori M, McDonald MV, Barrón Y, Cato K, Sockolow P, Sridharan S, Onorato N, Bowles K, Topaz M (2021). Improving patient prioritization during hospital-homecare transition: protocol for a mixed methods study of a clinical decision support tool implementation. JMIR Res Protoc.

[12] Vasquez MS (2009). Preventing rehospitalization through effective home health nursing care. Prof Case Manag.

[13] Murtaugh CM, Deb P, Zhu C, Peng TR, Barrón Y, Shah S, Moore SM, Bowles KH, Kalman J, Feldman PH, Siu AL (2017). Reducing readmissions among heart failure patients discharged to home health care: effectiveness of early and intensive nursing services and early physician follow-up. Health Serv Res.

[14] Topaz M, Trifilio M, Maloney D, Bar-Bachar O, Bowles KH (2018). Improving patient prioritization during hospital-homecare transition: a pilot study of a clinical decision support tool. Res Nurs Health.

[15] O'Connor M, Hanlon A, Bowles KH (2014). Impact of frontloading of skilled nursing visits on the incidence of 30-day hospital readmission. Geriatr Nurs.

[16] Markley J, Sabharwal K, Wang Z, Bigbee C, Whitmire L (2012). A community-wide quality improvement project on patient care transitions reduces 30-day hospital readmissions from home health agencies. Home Healthc Nurse.

[17] Deb P, Murtaugh C, Bowles K, Mikkelsen M, Khajavi H, Moore S, Barrón Y, Feldman P (2019). Does early follow-up improve the outcomes of sepsis survivors discharged to home health care?. Med Care.

[18] Sockolow P, Wojciechowicz C, Holmberg A, Bass EJ, Potashnik S, Yang Y, Bowles KH (2018). Home care admission information: what nurses need and what nurses have. A mixed methods study. Stud Health Technol Inform.

[19] Sockolow P, Bass EJ, Eberle CL, Bowles KH (2016). Homecare nurses' decision-making during admission care planning. Stud Health Technol Inform.

[20] Irani E, Hirschman KB, Cacchione PZ, Bowles KH (2018). Home health nurse decision-making regarding visit intensity planning for newly admitted patients: a qualitative descriptive study. Home Health Care Serv Q.

[21] (2011). State operations manual appendix B: guidance to surveyors home health agencies. Centers for Medicare & Medicaid Services.

[22] Nuccio E, Richard A (2010). Do delays in initiation of home healthcare services following hospital discharge affect patient outcomes?. Home Healthc Nurse.

[23] Morganti KG, Bauhoff S, Blanchard JC, Abir M, Iyer N, Smith A, Vesely JV, Okeke EN, Kellermann AL (2013). The evolving role of emergency departments in the United States. Rand Health Q.

[24] Fu S, Chen D, He H, Liu S, Moon S, Peterson K, Shen F, Wang L, Wang Y, Wen A (2021). Clinical concept extraction: a methodology review. J Biomed Inform.

[25] Topaz M, Murga L, Gaddis K, McDonald MV, Bar-Bachar O, Goldberg Y, Bowles KH (2019). Mining fall-related information in clinical notes: comparison of rule-based and novel word embedding-based machine learning approaches. J Biomed Inform.

[26] Topaz M, Adams V, Wilson P, Woo K, Ryvicker M (2020). Free-text documentation of dementia symptoms in home healthcare: a natural language processing study. Gerontol Geriatr Med.

[27] Topaz M, Woo K, Ryvicker M, Zolnoori M, Cato K (2020). Home healthcare clinical notes predict patient hospitalization and emergency department visits. Nurs Res.

[28] O'Connor M, Davitt JK (2012). The Outcome and Assessment Information Set (OASIS): a review of validity and reliability. Home Health Care Serv Q.

[29] Bui DDA, Zeng-Treitler Q (2014). Learning regular expressions for clinical text classification. J Am Med Inform Assoc.

[30] Gisev N, Bell J, Chen T (2013). Interrater agreement and interrater reliability: key concepts, approaches, and applications. Res Social Adm Pharm.

[31] O'Connor M, Bowles K, Feldman P, St Pierre M, Jarrín O, Shah S, Murtaugh CM (2014). Frontloading and intensity of skilled home health visits: a state of the science. Home Health Care Serv Q.

[32] Patient Safety Network (2019). Readmissions and adverse events after discharge. PSNet.

[33] Wang Y, Wang L, Rastegar-Mojarad M, Moon S, Shen F, Afzal N, Liu S, Zeng Y, Mehrabi S, Sohn S, Liu H (2018). Clinical information extraction applications: a literature review. J Biomed Inform.

[34] Sockolow P, Bowles K, Wojciechowicz C, Bass E (2020). Incorporating home healthcare nurses' admission information needs to inform data standards. J Am Med Inform Assoc.

[35] Bowles KH, Pham J, O'Connor M, Horowitz DA (2010). Information deficits in home care: a barrier to evidence-based disease management. Home Health Care Manag Pract.

[36] Jones C, Jones J, Richard A, Bowles K, Lahoff D, Boxer R, Masoudi F, Coleman E, Wald H (2017). "Connecting the dots": a qualitative study of home health nurse perspectives on coordinating care for recently discharged patients. J Gen Intern Med.

[37] Berland A, Bentsen S (2017). Medication errors in home care: a qualitative focus group study. J Clin Nurs.

[38] Hellesø R, Lorensen M, Sorensen L (2004). Challenging the information gap--the patients transfer from hospital to home health care. Int J Med Inform.

[39] Brody AA, Gibson B, Tresner-Kirsch D, Kramer H, Thraen I, Coarr ME, Rupper R (2016). High prevalence of medication discrepancies between home health referrals and Centers for Medicare and Medicaid Services home health certification and plan of care and their potential to affect safety of vulnerable elderly adults. J Am Geriatr Soc.

[40] Jones C, Jones J, Bowles K, Flynn L, Masoudi F, Coleman E, Levy C, Boxer R (2019). Quality of hospital communication and patient preparation for home health care: results From a statewide survey of home health care nurses and staff. J Am Med Dir Assoc.

[41] Arbaje A, Hughes A, Werner N, Carl K, Hohl D, Jones K, Bowles K, Chan K, Leff B, Gurses A (2019). Information management goals and process failures during home visits for middle-aged and older adults receiving skilled home healthcare services after hospital discharge: a multisite, qualitative study. BMJ Qual Saf.

[42] Romagnoli KM, Handler SM, Ligons FM, Hochheiser H (2013). Home-care nurses' perceptions of unmet information needs and communication difficulties of older patients in the immediate post-hospital discharge period. BMJ Qual Saf.

[43] Socio-behavioral determinants of health (SBDH) data catalog. John Hopkins Institute for Clinical & Translational Research.

[44] Li J, Qi M, Werner RM (2020). Assessment of receipt of the first home health care visit after hospital discharge among older adults. JAMA Netw Open.

