# Supplementary material for: Exploring Reasons for Delayed Start-of-Care Nursing Visits in Home Health Care: Algorithm Development and Data Science Study
Source: JMIR Nurs. 2021 Dec 30;4(4):e31038. doi: 10.2196/31038 (PMC8759020; doi:10.2196/31038)
Supplement: Multimedia Appendix 1 [file nursing_v4i4e31038_app1.docx]

Appendix 1- Examples of regular expression rules developed to identify reasons for delayed start-of-care HHC nursing visits in clinical notes.

| Category | Example of clinical notes with language indicative of delayed start-of-care | Example of regular expression rules. Bold font indicates parts of the formulated regular expression rules that are able to recognize the pattern of delayed start-of-care in the clinical notes (mentioned in the previous column) |
| --- | --- | --- |
| No answer at the door or phone | SOC [abbreviation: start of care] ATTEMPTED, **UNABLE** TO **LEAVE VM** [abbreviation: voice message] TO CONFIRM VISIT, MAILBOX FULL**UNABLE** TO LEAVE **MSG** | (doesnt\|wrong\|no\|not\|**unable**\|incorrect\|cannot\|doesnot)(?:\W+\w+)**{0,3}?\W+(**answer\w*\|voicemail\|voice mail\w*\|voice\s*\|**vm**\|**msg**\|messag\w*\|response\|phone\|number\|address)\b", str(row)) |
| Patient/family request to postpone or refuse some HHC services | Nurses contacted the patient, “**Patient** replied but **declined SOC** [abbreviation: start of care] today” | (**?!**(refus\w*\|declin\w*)(?:\W+\w+){0,3}?\W+(function\w*\|homecare\s+reason\w*\|homecare\s+diagnosis))(?:(refus\w*\|**declin**\w*)(?:\W+\w+){0,3}?\W+(servic\w*\|vns\|homecar\w*\|visit\w*\|care\|**soc**\|chaa\|vnsny) |
|  | “Caregiver and daughter informed VN [abbreviation: visiting nurse] that pt [abbreviation: patient] will not be available today and made the **request** to **reschedule SOC** for **tomorrow”** | (?!(^(request\w* soc\|request\w* start of care)))  (?!(request\w* start of care:\|request\w* soc:))  (?:(want\w*\|**reques**\w*\|prefer\w*\|prfer\w*\|prefr\w*\|cancel\w*\|ask\|would like\|will like)  (?:\W+\w+){0,3}?\W+(visi\w*\|**soc**\|eval\w*\|hospice\|  vnschoice\|hcc\|vns_svcs\|start of care)(?:\W+\w+){0,2}?\W+(\d{4}  (\/\|-\|\.)\d{1,2}(\/\|-\|\.)\d{1,2}\|\d{1,2}(\/\|-\|\.)\d{1,2}(\/\|-\|\.)\d{4}\|\d{1,2}(\/\|-\|\.)\d{1,2}\|\d{1,2}  \|jan\|feb\|mar\|apr\|may\|jun\|jul\|aug\|sep\|oct\|nov\|dec\|monday\|mon*\|tues\w*\|  wednes\w*\|tursd\w*\|frid\w*\|weekend\|sunday\|satur\w*\|  **tomorrow\|tomorow**\|tomor\w*\|week\|next week\|next day\|day\|next))) |
| Administrative or scheduling issues | “Request for VN [abbreviation: visiting nurse] visit, **FFE** was **not** **obtained**.” | (**ffe**)**(?:\W+\w+){0,3}**?\W+(not\|non\|no\|doesnot\|doesn't)(?:\W+\w+){0,3}?\W+(provid\w*\|**obtain**\w*\|admit\w*\|attach\w*) |
